# Pharmacological Modulation of Melanocortin 1 Receptor Signaling by Mrap Proteins in *Xenopus tropicalis*

**DOI:** 10.3389/fendo.2022.892407

**Published:** 2022-06-20

**Authors:** Xiaolu Tai, Yaqun Zhang, Jindong Yao, Xuan Li, Jun Liu, Jiazhen Han, Jianjun Lyu, Gufa Lin, Chao Zhang

**Affiliations:** ^1^ Fundamental Research Center, Shanghai YangZhi Rehabilitation Hospital (Shanghai Sunshine Rehabilitation Center), School of Life Sciences and Technology, Tongji University, Shanghai, China; ^2^ Department of Pathology, InnoStar Bio-tech Nantong Co., Ltd., Nantong, China; ^3^ Key Laboratory of Spine and Spinal Cord Injury Repair and Regeneration of Ministry of Education, Orthopaedic Department of Tongji Hospital, School of Life Sciences and Technology, Tongji University, Shanghai, China

**Keywords:** *Xenopus tropicalis*, amphibian, Mc1r, Mrap1, Mrap2

## Abstract

The melanocortin system consists of five G protein–coupled receptors (MC1R-MC5R), the bidirectional endogenous ligands (MSH and Agouti families), and accessory proteins (MRAP1 and MRAP2). Accumulative studies of vertebrate species find high expression level of melanocortin 1 receptor (MC1R) in the dermal melanocyte and elucidate the essential roles in the skin and fur pigmentation, morphological background adaptation, and stress response. The diploid amphibian *Xenopus tropicalis* (*xt*) has been utilized as a fantastic animal model for embryonic development and studies of physiological cryptic colouring and environmental adaptiveness. However, the interaction of xtMc1r signaling with xtMrap proteins has not been assessed yet. In this study, we carried out *in silico* evolutionary analysis of protein alignment and genetic phylogenetic and genomic synteny of *mc1r* among various vertebrates. Ubiquitous expression of *mrap1* and *mrap2* and the co-expression with *mc1r* transcripts in the skin were clearly observed. Co-immunoprecipitation (ip) and fluorescent complementary approach validated the direct functional interaction of xtMc1r with xtMrap1 or xtMrap2 proteins on the plasma membrane. Pharmacological assay showed the improvement of the constitutive activity and alpha melanocyte-stimulating hormone (α-MSH) stimulated plateau without dramatic alteration of the cell surface translocation of xtMc1r in the presence of xtMrap proteins. Overall, the pharmacological modulation of xtMc1r by dual xtMrap2 proteins elucidated the potential role of this protein complex in the regulation of proper dermal function in amphibian species.

## Introduction

The melanocortin system regulates broad physiological functions in mammals and some other vertebrate species. It consists of five rhodopsin-like G protein–coupled receptors (melanocortin receptor, MC1R–MC5R), the endogenous bidirectional agonistic and antagonistic ligand pairs (melanocyte stimulating hormone, MSH, and Agouti protein families) and dimeric single transmembrane melanocortin receptor accessory proteins (MRAP1 and MRAP2) (1–5). The MC1R is previously found in the dermal melanocytes where its activation by α-MSH elevates downstream tyrosinase cascades and stimulates eumelanin pigmentation of hair follicles in multiple mammalian species (6–8). Intriguingly, insights from large mammals suggests that the constitutive activation of MC1R is more important for dark hair or coat color, whereas α-MSH–induced MC1R signaling plays a key role in increases of eumelanin production and causes skin tanning (9). The endogenous antagonist Agouti of MC1R locally blocks the effect of α-MSH and promotes pheomelanin expression in the fur (10, 11). Ubiquitous ectopic over-expression of Agouti leads to a yellow coat color and obesity syndrome in murine model (12).

In lower vertebrates, such as teleosts and amphibians, the chronic dermal pigmentation is controlled by the developmental differentiation of chromatoblast into various combinations of melanophore, xanthophore, iridophore, cyanophore, etc. (13–15). As a camouflage mechanism, the acute morphological skin color change is bidirectionally regulated by aggregation or dispersion of intracellular melanin. This physiological response is caused by neuronal derived luminescent stimuli from the surroundings to aid for the hiding and prevention of superior predators and is simultaneously regulated by α-MSH-Mc1r and MCH (melanin-concentrating hormone)–Mchr (melanin-concentrating hormone receptor) signaling pathways (16–21). We previously demonstrated the requirement of both eyes and pineal glands for the teleostean background adaptation. In the zebrafish, pineal AgRP2 neurons projected to the lateral tubular nucleus, specifically antagonized zMc1r signaling and stimulated the hypothalamic synthesis of two melanin concentrating hormones (MCH and MCH-like) (21). Moreover, chordate Mc1r signaling is regulated by Mrap proteins in red stingray (Dasyatis akajei) (22) and orange-spotted grouper (Epinephelus coioides) (23) and is strongly associated with the stress response in the rainbow trout (Oncorhynchus mykiss) (24) and zebrafish (Danio rerio) (25).

*Xenopus tropicalis* serves as an ideal model system for the skin pigmentation and background adaptation for decades due to the stronger adaptative capability than teleosts (26, 27). α-MSH and Agouti, the natural Mc1r ligands, are reported to modulate pigment-type switching in *Xenopus* melanophores (28, 29). Mrap protein family functions as vital molecular chaperones for all melaocortin receptors (30). Recently, the pharmacological modulation and physiological roles of dual melanocortin accessory proteins (Mrap1 and Mrap2) on the modulation of Mc2r, Mc3r, and Mc4r signaling have been examined in the *Xenopus tropicalis* and *Xenopus laevis* (31–34). However, the potential physiological correlation of Mc1r with Mrap proteins has not been fully investigated yet. In this study, we carried out multiple *in silico* and biochemical approaches to explore the evolutionary aspect of *Xenopus tropicalis* Mc1r (xtMc1r) and elucidated the protein interaction and pharmacological correlation with two Mrap proteins (xtMrap1 and xtMrap2) *in vitro*. This is the first evaluation of the pharmacological profile of melanocorin accessory proteins on modulating Mc1r signaling in diploid amphibian species.

## Methods and Materials

### Plasmids

The nucleic acid and amino acid sequences of *mc1r*, *mrap1*, and *mrap2* were acquired from the National Center for Biotechnology Information database and our previous study (32). The DNA fragments of these genes were amplified from the brain cDNA library of an adult female *Xenopus tropicalis* and sub-cloned into pcDNA3.1 (+) vector with proper epitope tags at both N- and C-terminals. All the sequences were verified by DNA sequencing (Genewiz from Azenta Life Sciences, China).

### Protein Alignments, Phylogenetics, and Genomic Synteny Analysis

We performed *in silico* online Multiple Sequence ClustalW Alignment (https://www.genome.jp/tools-bin/clustalw) with default parameters. The phylogenetic trees of *mc1r* from various vertebrates were calculated and generated by MEGA5.1 software, and the analysis for *mrap1* and *mrap2* was previously reported (32). The genomic synteny and comparative analysis of the adjacent genomic regions of *mc1r* in elephant shark, zebrafish, *Xenopus tropicalis*, turtle, chicken, mouse, and human was carried out on the UCSC genome browser (http://genome.ucsc.edu/).

### Tissue Distribution Analysis of xtmc1r and xtmraps

An adult female *Xenopus tropicalis* (1 year old) was euthanized with overdose of Tricaine methane sulfonate (MS‐222) with the approved protocol by the Institutional Animal Care and Use Committee (IACUC) of Tongji University. The total RNA of each tissue was extracted with TRNzol Universal Reagent (TIANGEN Biotech, Beijing, China) and reverse-transcribed with the FastKing RT Kit (KR116, TIANGEN Biotech, Beijing, China) to remove the residual genomic DNA contaminations. Next, the cDNA was utilized for the following tissue distribution analysis as previously reported (32). The *β-actin* was used as an internal control. qPCR primers are as follows: xtMC1R_fw CCCACATCAAGCTAGGGCAA; xtMC1R_rev GCCATTAGCTTTCTGCCAGC; xtMRAP1_fw GGCACTAGCTCTGCTCACAA; xtMRAP1_rev ACAAACATTGCAAGGCCGAC; xtMRAP2_fw TGGGTTGGTCTTGCAGTCTT; xtMRAP2_rev TCCTTCCAAAATCAGGCGCA; xtβ-actin_fw AACCGGGAGAAAATGACGCA; xtβ-actin_rev ACAGGGACAACACAGCTTGG. The RT-PCR and agarose gel analysis were repeated three times for each gene.

### Western Blot and Co-Immunoprecipitation Assay

The HEK293T cells were cultured with high glucose dulbecco’s modified eagle medium (DMEM) containing 10% (v/v) fetal bovine serum (Gibco, Australia) in an incubator with 5% CO_2_ at 37°C. Plasmids with proper genes or empty vectors were transfected with polyethyleneimine (PEI, Polysciences, Inc., USA). 3×HA-xtMc1r and v5-xtmraps-flag were co-transfected, lysed, and incubated with HA-Tag (C29F4) Rabbit mAb (Cell Signaling Technology, Inc., USA) overnight at 4°C. The next day, protein A+G agarose beads (Beyotime Institute of Biotechnology, Shanghai, China) were applied, washed, and re-suspended in native sample loading buffer with β-mercaptoethanol (Sangon Biotech Ltd., Shanghai, China). Samples were boiled at 95°C for 15 min, resolved with sodium dodecyl sulfate – polyacrylamide gel electrophoresis (SDS/PAGE) gel, and transferred to the polyvinylidene difluoride (PVDF) membranes (Millipore Sigma Chemicals, Fisher Scientific, USA). Next the membranes were shaken with Immunol Staining Blocking Buffer with BSA and Triton X-100 (P0102, Beyotime Institute of Biotechnology, Shanghai, China) for 15 min and then incubated with 1:2,000 diluted HA-tag Rabbit mAb (Cell Signaling Technology, Inc., USA), Mouse anti FLAG-tag mAb (ABclonal Biotechnology Co., Ltd., China), at 4°C overnight. Next day, the secondary peroxidase Horseradish Peroxidase (HRP)-conjugated antibody (ABclonal Biotechnology Co., Ltd., China) was diluted by 1:4,000 and applied for extra 2 h at room temperature. Finally, staining of the membranes was exposed to the enhanced chemiluminescence plus reagents (Beyotime Institute of Biotechnology, Shanghai, China), and the images were captured by the Amersham Imager 600 (GE Healthcare Life Sciences, USA).

### Bimolecular Fluorescent Complementation Assay

The Venus fluorescent protein was divided into two non-fluorescent fragments, VF1 and VF2, as previously reported (32). HEK293T cells were cultured in poly-d-lysine–coated plates (Sangon Biotech Ltd., Shanghai, China) and transfected with polyethyleneimine. Plates were washed with phosphate buffer solution and fixed with 4% (w/v) paraformaldehyde (Sangon Biotech Ltd., Shanghai, China) after 24 h transfection. Cells were then permeabilized by applying 0.3% (v/v) Tween 20 and 5% (v/v) goat serum and incubated overnight with 1:2,000 diluted FLAG-tag Mouse mAb (Cell Signaling Technology, Inc., USA). Plates were then washed three times with phosphate buffered saline (PBS) and incubated with 1:1,000 diluted Goat Anti-Rabbit immunoglobulin G (IgG) (Alexa Fluor 555) antibody (Abcam plc., UK) for 2 h at room temperature. Finally, Gold Antifade Reagent with 4',6-diamidino-2-phenylindole (DAPI) (Cell Signaling Technology, Inc., USA) was applied to stain cell nuclei, and then the plates were sealed with coverslips. The fluorescence excitation was captured by the Zeiss LSM880 AiryScan Confocal Microscope (Jena, Germany) with 60× oil immersion lens.

### cAMP Luciferase Reporter Assay

The *mc1r* and *mraps* plasmids were transiently co-transfected at 1:0, 1:1, 1:3, and 1:6, along with the pCRE-luc reporter vector in HEK293T cells. DMEM with 0.1% bovine serum albumin (Sangon Biotech Ltd., Shanghai, China) was utilized to dilute α-MSH peptide (GenScript ProBio, China) from 10^−6^ to 10^−10^. Cells were distributed equally into 24-well plate and then incubated with corresponding concentrations of α-MSH for 4 h at 37°C. For the antagonistic assay, cells were incubated with EC_80_ dosage of α-MSH with different dilutions of AgRP peptide (GenScript ProBio, China). The luminescence was excited with Dual-Glo Luciferase Assay Reagent System (Promega Biotech Co., Ltd., USA) and measured by Spectramax iD3 Multi-Mode Microplate reader. Each assay was performed and replicated in triplicate for statistical analysis.

### Cell-Surface Enzyme-Linked Immunosorbent Assay

HEK293T cells were cultured in poly-d-lysine–pre-coated 24-well plates and transfected with *mc1r* and *mraps* plasmids at ratios of 1:0, 1:1, 1:3, and 1:6. Twenty-four hours later, plates were washed with PBS and fixed with 4% (w/v) paraformaldehyde (Sangon Biotech Ltd., Shanghai, China). Cells were then incubated with 5% (m/v) non-fat milk (Sangon Biotech Ltd., Shanghai, China) in Dulbecco's Phosphate Buffered Saline (DPBS) for 1 h at room temperature and incubated with 1:4,000 diluted HA-tag Rabbit mAbs (Cell Signaling Technology, Inc., USA) for 2 h. Cell were washed with DPBS and incubated with 1:7,500 diluted secondary peroxidase (HRP)–conjugated antibody (ABclonal Biotech, China) for 1 h. Finally, the TMB substrate solution (Beyotime Institute of Biotechnology, Shanghai, China) was applied and stopped by adding 2 M H_2_SO_4_. The luminescent signal was detected and measured at OD_450_ by Spectramax iD3 Multi-Mode Microplate reader. Each assay was performed and replicated in triplicate for the following statistical analysis.

### Statistical Analysis

The raw data generated from above were analyzed by GraphPad Prism 6 (https://www.graphpad.com/). Statistical differences between experimental conditions and control groups were analyzed with one-way ANOVA and Tukey post-test, whereas two independent groups were compared by the Student’s *t*-test with a significance level of 0.05. Not significant (ns), ∗p < 0.05, ∗∗p < 0.01, ∗∗∗p < 0.001, and ∗∗∗∗p < 0.0001. Data were plotted as standard error of mean ± SEM. All experiments were replicated in triplicate and repeated at least three separate times.

## Results

### Evolutionary Conservation and Tissue Distribution Analysis of *mc1r*

**Figure 1:**
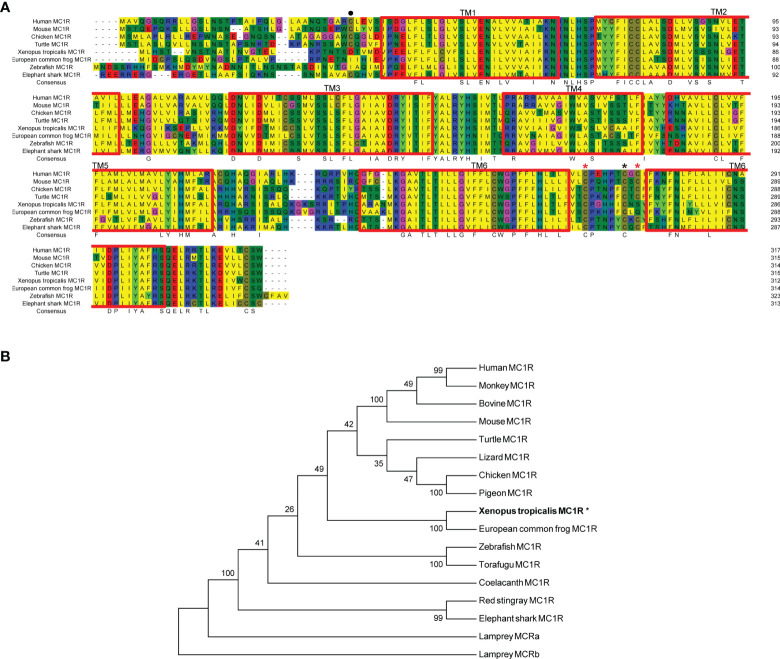
Protein alignment and phylogenetic analysis of *Xenopus tropicalis* Mc1r. **(A)** Sequence alignments of xtMc1r (XP_012817790.1) and other Mc1rs from human (NP_002377.4), mouse (NP_032585.2), monkey (NP_032585.2), bovine (NP_776533.1), chicken (NP_001026633.1), pigeon (OPJ78282.1), turtle (XP_005308247.1), European common frog (ACA28876.1); zebrafish (NP_851301.1), torafugu (AAO65548.1), coelacanth (XP_005999265.1), red stingray (BAU98230.1), elephant shark (ENSCMIT00000036457.1), lamprey Mca receptor (XP_032816350.1), and lamprey Mcb receptor (ABB36647.1).The blue, red, and yellow represent a homology over 50%, 75%, and 100%, respectively. **(B)** Dendrogram of Mc1rs was generated by the NJ analysis with Molecular Evolutionary Genetics Analysis (MEGA) software. Asterisk (*) indicates xtMC1R with bold letters.

**Figure 2:**
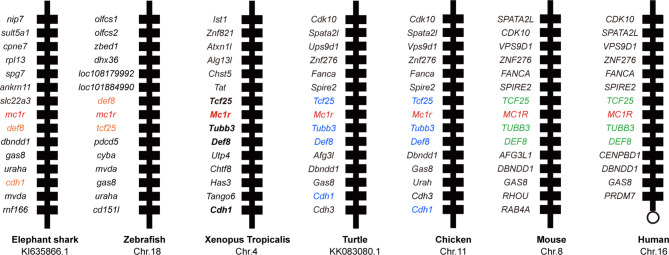
Synteny analysis of *Xenopus tropicalis mc1r*. Synteny mapping of *mc1rs* among with *Callorhinchus milii* (elephant shark), *Danio rerio* (zebrafish), *Xenopus tropicalis*, *Chrysemys picta bellii* (turtle), *Gallus gallus* (chicken), *Mus musculus* (house mouse), and *Homo sapiens* (human). Positional conserved genes among multiple species are marked with color.

**Figure 3:**
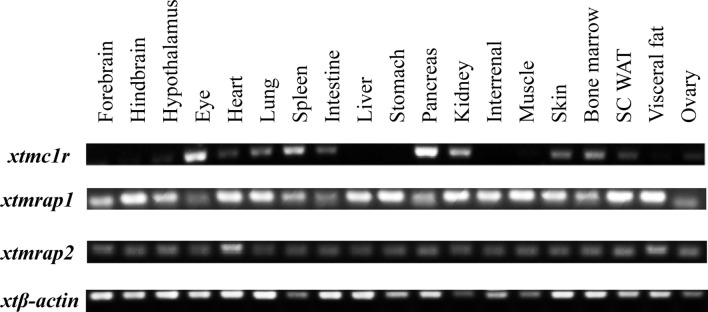
Expressional analysis of *mc1r* transcript in multiple tissues of *Xenopus tropicalis*. Expression profiles of *mc1r*, *mrap2*, and *mrap2* transcript in 19 tissues from an adult female *Xenopus tropicalis*. Housekeeping gene *β-actin* was used as an internal control.

First we picked *mc1r* genes from eight vertebrates including two mammals (human and mouse), one bird (chicken), one reptile (green Western Painted Turtle), two amphibians (*Xenopus tropicalis* and *European common frog*), and two fishes (zebrafish and elephant shark) and performed the protein sequence alignment analysis (**Figure 1A**). Overall, the transmembrane domains (TMs 1–7), the first and second intracellular loop, and C terminal were highly conserved, and the whole MC1R protein showed only 30.3% primary sequence identity in all vertebrates. The *Xenopus tropicalis* Mc1r showed highest 62.8% similarity with *European common frog* and lowest 51.4% similarity with human ortholog. Next, we analyzed and generated the phylogenetic tree of all *mc1rs* and found that two amphibians clustered into a separated clade as shown in the dendrogram (**Figure 1B**). In addition, with the available UCSC genome browser database, we performed genomic synteny and monitored the arrangement of surrounding genomic regions of *mc1r* in elephant shark, zebrafish, *Xenopus tropicalis*, Western Painted Turtle, chicken, mouse, and human and illustrated the positional orders of key adjacent genes of *Xenopus tropicalis mc1r*, in which three genes including *tcf25*, *tubb3*, and *def8* were in strict accordance with mammalian species (**Figure 2**). Next, we dissected an adult female *Xenopus tropicalis* and examined the transcripts of *mc1r* and *mraps* by reverse-transcription PCR (RT-PCR) in 19 tissues. We found that the *mc1r* and *mrap2* exhibited ubiquitous expressional pattern and high level of *mc1r* transcript was seen in the skin, eye, spleen, pancreas, kidney, and bone marrow (**Figure 3**). In summary, both genetic phylogeny and genomic synteny investigation clarified the evolutionary conservation of *mc1r* in vertebrates. The co-existence of *mc1r* and *mraps* transcripts in the skin and some other organs strongly suggested the potential coordination and co-participation in the dermal and some other unknown physiological functions.

### Co-Localization and Direct Interaction of xtMc1r and xtMrap Proteins

**Figure 4:**
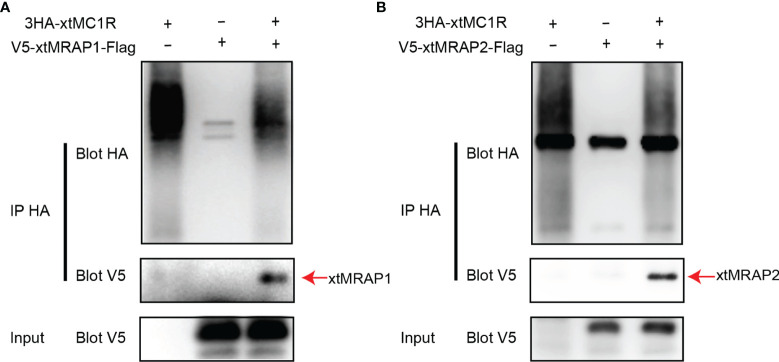
Investigation of the direct Protein interaction of xtMraps and xtMc1r proteins *in vitro*. **(A)** Co-immunoprecipitation of the HA-xtMc1r and Flag-xtMrap1 protein complex. **(B)** Co-immunoprecipitation of HA-xtMc1r and Flag-xtMrap2 protein complex.

**Figure 5:**
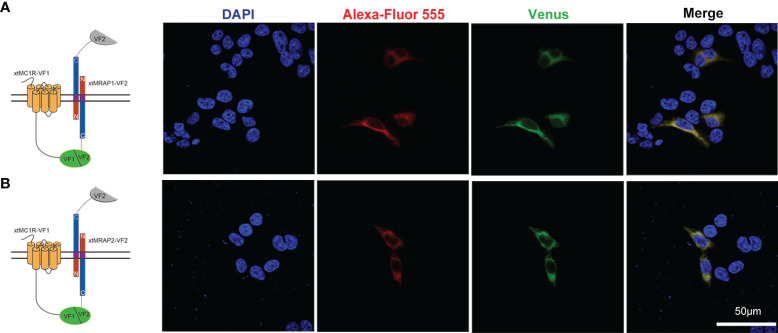
Functional protein complex of xtMc1r and xtMraps on plasma membrane. **(A)** Formation of functional protein complex of xtMc1r and xtMrap1 on the plasma membrane. **(B)** Formation of functional protein complex of xtMc1r and xtMrap2 on the plasma membrane. Nuclei were shown in blue (DAPI). Scale bar = 50 μm.

3HA-xtMc1r and v5-xtMrap1-flag or v5-xtMrap2-flag were co-transfected into HEK293T cells, and a tight protein complex was steadily observed by co-immunoprecipitation *in vitro* (**Figure 4**). Next, we performed the bimolecular fluorescence complementation assay to validate the actual and functional xtMc1r-xtMraps complex in live cells. The complementary Venus signal was clearly captured on the plasma membrane and several intracellular compartments (**Figure 5**) suggesting that the non-fluorescent fragments of VF1 and VF2 physically became close to each other. Overall, our experimental evidence confirmed the actual direct protein interactions of xtMraps and xtMc1r.

### Pharmacological Effect of xtMraps on Modulating xtMc1r Signaling

**Figure 6:**
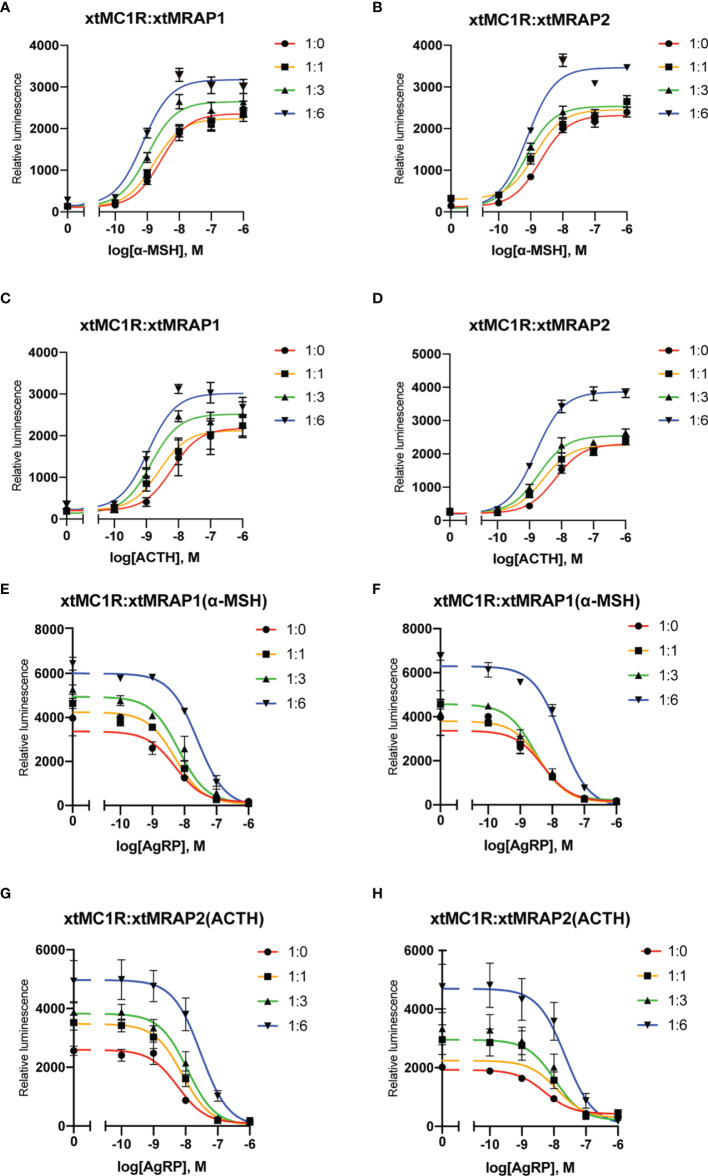
Pharmacological modulation of xtMc1r signaling by xtMrap proteins. **(A–D)** Dose-responsive cAMP level of α-MSH (0 M, 10^−11^ to 10^−6^ M) and ACTH (0 M, 10^−11^ to 10^−6^ M) stimulated xtMc1r in presence of different amounts of xtMrap1**(A, C)** and xtMrap2 **(B, D)**. Data were represented as the mean ± SEM from three independent experiments **(E–H)**. The antagonistic effect of AgRP (10^−11^ to 10^−6^ M) to the EC_80_ dosage of α-MSH **(E, F)** or ACTH **(G, H)** induced xtMc1r signaling in presence of different amounts of xtMrap1**(E, G)** or xtMrap2 **(F, H)**. Data were represented as the mean ± SEM from three independent experiments.

**Figure 7:**
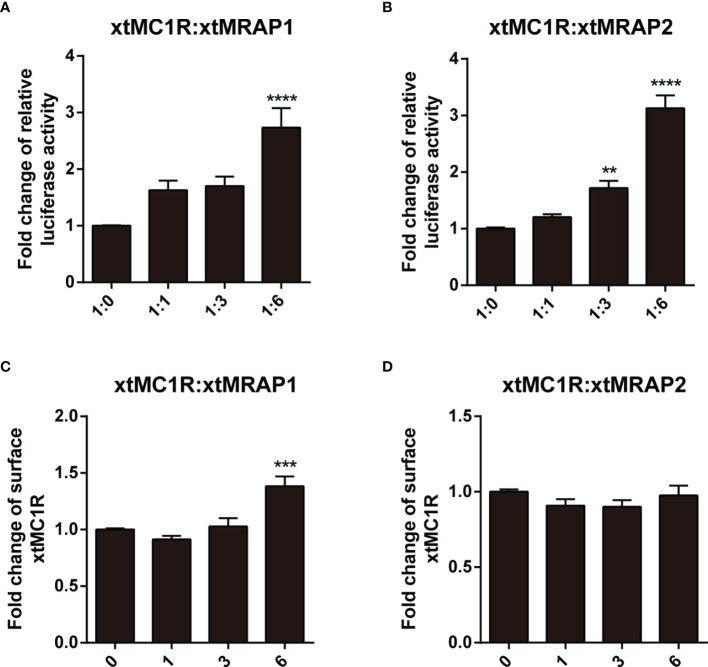
Measurement of the constitutive activity and surface translocation of xtMcar by xtMrap proteins. The constitutive activity of xtMc1r in the presence of xtMrap1 **(A)** or xtMrap2 **(B)** at ratio of 1:0, 1:1, 1:3, and 1:6. Surface expression level of the HA-tagged xtMc1r in the presence of xtMrap1 **(C)** or xtMrap2 **(D)** at ratio of 1:0, 1:1, 1:3, and 1:6. Data were represented as the mean ± SEM from three independent experiments. **p < 0.01, ^∗∗∗^p < 0.001 and ^∗∗∗∗^p < 0.0001.

Next, we evaluated the pharmacological profile of xtMc1r in the presence of xtMrap proteins. Our results showed that xtMrap1 and xtMrap2 dramatically elevated the constitutive activities of xtMc1r (**Figures 7A, B**). xtMc1r showed elevated α-MSH stimulated plateau with 1:6 ratios of xtMrap1 or xtMrap2 (**Figure 6** and **Table 1**). Next, we assessed the inhibitory effect of AgRP peptide on xtMc1r signaling in presence of EC_80_ of α-MSH or ACTH. xtMrap1 and xtMrap2 could dose-dependently suppress the AgRP-reduced xtMc1r signaling (**Figures 6C, D**). Moreover, EC_50_ of each curve was calculated and obviously altered in the presence of xtMrap1 or xtMrap2, suggesting that xtMrap proteins could affect the sensitivity of xtMc1r to α-MSH or AgRP. Together, xtMrap proteins exhibited dose-dependent potentiation of cAMP responsive plateau of xtMc1r signaling.

**Table 1:** Pharmacological summary of α-MSH and ACTH activated xtMc1r signaling in presence of different amounts of xtMrap proteins.

		1:0	1:1	1:3	1:6
xtMc1r:xtMrap1(α-MSH)	EC_50_ (M)	2.806 × 10^−9^ ± 3.992 × 10^−10^	1.429 × 10^−9^ ± 3.589 × 10^−10^*	9.662×10^−10^ ± 2.345×10^−10^**	6.906×10^−10^ ± 5.008 × 10^−11^***
xtMc1r:xtMrap1(ACTH)	EC_50_ (M)	7.006 × 10^−9^ ± 8.413 × 10^−10^	3.309 × 10^−9^ ± 6.033 × 10^−10^***	1.171 × 10^−9^ ± 2.343 × 10^−10^****	1.185 × 10^−9^ ± 1.030 × 10^−10^****
xtMc1r:xtMrap2(α-MSH)	EC_50_ (M)	2.036 × 10^−9^ ± 1.632 × 10^−10^	1.110 × 10^−9^ ± 3.419 × 10^−10^*	7.550 × 10^−10^ ± 1.226 × 10^−10^**	7.799 × 10^−10^ ± 4.374 × 10^−11^**
xtMc1r:xtMrap2(ACTH)	EC_50_ (M)	7.150 × 10^−9^ ± 1.198 × 10^−9^	4.104 × 10^−9^ ± 1.246 × 10^−9^	2.885 × 10^−9^ ± 9.632 × 10^−10^*	1.513 × 10^−9^ ± 1.268 × 10^−10^**
xtMc1r: xtMrap1 (AgRP-1 × 10^−8^ M α-MSH)	IC_50_ (M)	3.400 × 10^−9^ ± 2.415 × 10^−9^	6.228 × 10^−9^ ± 1.846 × 10^−9^	1.188 × 10^−8^ ± 6.229 × 10^−9^	2.318 × 10^−8^ ± 1.375 × 10^−9^*
xtMc1r: xtMrap1 (AgRP-2 × 10^−8^ M ACTH)	IC_50_ (M)	3.738 × 10^−9^ ± 2.483 × 10^−9^	3.777 × 10^−9^ ± 2.710 × 10^−10^	1.145 × 10^−8^ ± 6.068 × 10^−9^	2.884 × 10^−8^ ± 5.360 × 10^−9^*
xtMc1r: xtMrap1 (AgRP-1 × 10^−8^ M α−MSH)	IC_50_ (M)	3.400 × 10^−9^ ± 2.415 × 10^−9^	2.225 × 10^−9^ ± 1.214 × 10^−9^	5.438 × 10^−9^ ± 2.393 × 10^−9^	1.941 × 10^−8^ ± 8.700 × 10^−10^**
xtMc1r: xtMrap1 (AgRP-2 × 10^−8^ M ACTH)	IC_50_ (M)	4.398 × 10^−9^ ± 3.860 × 10^−10^	3.968 × 10^−9^ ± 4.700 × 10^−11^	4.932 × 10^−9^ ± 1.690 × 10^−10^	2.010 × 10^−8^ ± 2.030 × 10^−9^***

Data were represented as the mean ± SEM from three independent experiments. *p < 0.05, **p < 0.01, ***p < 0.001, and ****p < 0.0001.

### Surface Translocation of xtMc1r in Presence of xtMrap Proteins

Next, we checked whether the cell surface translocation of xtMc1r could be affected by the presence of xtMrap proteins. 3HA-xtMc1r and v5-xtMraps-flag were co-transfected at ratios of 1:0, 1:1, 1:3, and 1:6 in HEK293T cells. Enzyme-linked immunosorbent assay was carried out to quantify the surface level of 3HA-tagged xtMc1r proteins. As shown, the surface expression of xtMc1r did not dramatically change in various dosages of xtMrap2 (**Figure 7D**) and only elevated with high dosage of xtMrap1 proteins (**Figure 7C**). Overall, these results showed that both xtMrap proteins exhibited mild effect on the cell surface translocation of xtMc1r.

## Discussion

The fur color of mammals is regulated by the synthesis of melanin in the melanocyte. As the first discovered melanocortin receptor, the activation of MC1R signaling by the locally secreted α-MSH in the skin is vital for the initiation of tyrosinase-associated biochemical reactions. In lower vertebrates, dermal pigmentation is determined by the controlled differentiation of chromatoblast into melanophore, xanthophore, iridophore, cyanophore, etc. The morphological cryptic skin color adaptation, a camouflage mechanism for animals’ hiding and prevention of natural predators, is simultaneously regulated by acute aggregation or dispersion of intracellular melanin aggregates triggered by luminescent signal from the surroundings. This vital physiological response is bidirectionally modulated by α-MSH-Mc1r and MCH-Mchr signaling pathways. *Xenopus tropicalis*, a diploid amphibious animal, serves as an ideal model system for the skin pigmentation and background adaptation studies for decades due to its stronger adaptative capability than teleostean species (26, 27). α-MSH and Agouti, the natural Mc1r ligands, could modulate pigment-type switching in *Xenopus* melanophores (28, 29).

Accumulated evidence found that the melanocortin system existed only in chordate phyla, and the most ancient melanocortin receptor was reported in the lamprey and hagfish genomes already (35, 36). Recently, the physiological roles of multiple melanocortin accessory proteins on the regulation of Mc2r, Mc3r, and Mc4r signaling have been elucidated in the diploid *Xenopus tropicalis* and tetraploid *Xenopus laevis* (30–34) without any attention to the Mc1r. In our previous study, we had performed the protein alignment, phylogenetic tree, and synteny analysis of *mrap1* and *mrap2*, along with *mc3r* and *mc4r* (32). In this study, with multiple bioinformatic and biochemical approaches, we explored the evolutionary aspect of *mc1r* and other orthologs among various vertebrates. The protein alignment, phylogenetic tree, and genomic synteny clearly verified the closest relative of the amphibians among eight vertebrates from selachian to mammals (**Figures 1** and **2**). Like mammals, the high mRNA expression of *mc1r* transcript was seen in the skin, eye, spleen, pancreas, kidney, and bone marrow. In accordance with our previous finding (32), we observed the ubiquitous expression of *mrap1* and *mrap2* in 19 collected tissues (**Figure 3**). Importantly, co-expression of three transcripts in the skin, the major region for exerting Mc1r function strongly indicated the association of Mrap proteins in the regulation of dermal function and Mc1r signaling.

As the naturally existing endogenous agonists, α-MSH or ACTH peptide could directly activate the downstream Gαs-coupled cAMP cascades of all melanocortin receptors, along with the accessory and regulatory effect from the Mrap protein families on the cell surface (2, 37–39). However, the pharmacological modulation of Mrap proteins on every melanocortin receptor signaling differs greatly among vertebrates. Here, in this study, we verified the direct protein interaction, the pharmacological modulation of two xtMrap proteins on the xtMc1r cascades upon activation by α-MSH or inhibition by AgRP, respectively (**Figures 4**–**6**). We also confirmed the actual existence of functional protein complex of xtMc1r-xtMrap1 and xtMc1r-xtMrap2 on the cell surface by venus fluorescence complementation approach (**Figure 5**). In addition, xtMrap1 and xtMrap2 significantly elevated the constitutive activities without affecting the cell surface translocation of xtMc1r (**Figure 7**).

In lower vertebrates, especially the teleosts, Mc1r mainly participates in the dermal melanin synthesis and melanophore dispersion (21, 22). Interestingly, the rainbow trout mc1r transcript is strongly associated with the stress response (24). The distribution of *mc1r* transcript in multiple organs indicates that it may participate in other unknown physiological functions in *Xenopus tropicalis.* Moreover, the angstrom resolution of cryo-EM structures of the human MC1R-Gs complexes have been reported recently (40). With this technique, the structural insights of Mrap-Mc1r-Gs complex of several species may be elucidated in the near future.

Together, we performed a comprehensive genetic and genomic analysis of the evolutionary and functional aspect of Mc1r with two accessory proteins in the *Xenopus tropicalis*. The elevation of the ligand stimulated maximal response of xtMc1r signaling suggested that the xtMrap proteins jointly participated in the multiple physiological processes in the skin. The ubiquitous expression of two *mrap* transcripts strongly indicated the broad distribution and functional diversity of Mrap proteins in the *Xenopus tropicalis* and impelled us to further elucidate the physiological and pharmacological regulation on other G protein-coupled receptor (GPCR)-associated pathways in the amphibian species.

## Data Availability Statement

The original contributions presented in the study are included in the article/supplementary material. Further inquiries can be directed to the corresponding authors.

## Ethics Statement

The animal study was reviewed and approved by Institutional Animal Care and Use Committee (IACUC) at the Tongji University.

## Author Contributions

XT, GL, and CZ participated in the design of the study. XT, YZ, JY, and XL performed the experiments. JL, JH, and JJL contributed to data collection and data analysis. XT and CZ contributed to the manuscript writing. All authors contributed to the article and approved the submitted version.

## Funding

The work was supported by grants from National Key Research and Development Program of China (Grant No. 2017YFA0103902), the Innovative Research Team of High-level Local Universities in Shanghai (Grant No. SSMU-ZDCX20180700), and a Key Laboratory Program of the Education Commission of Shanghai Municipality (Grant No. ZDSYS14005).

## Conflict of Interest

YZ, JL, and JJL are employed by InnoStar Bio-tech Nantong Co., Ltd.

The remaining authors declare that the research was conducted in the absence of any commercial or financial relationships that could be construed as a potential conflict of interest.

## Publisher’s Note

All claims expressed in this article are solely those of the authors and do not necessarily represent those of their affiliated organizations, or those of the publisher, the editors and the reviewers. Any product that may be evaluated in this article, or claim that may be made by its manufacturer, is not guaranteed or endorsed by the publisher.
